# Cancer Treatment With the Ketogenic Diet: A Systematic Review and Meta-analysis of Animal Studies

**DOI:** 10.3389/fnut.2021.594408

**Published:** 2021-06-09

**Authors:** Jing Li, Haiyan Zhang, Zhu Dai

**Affiliations:** Pharmaceutical Department, Hubei Cancer Hospital, Tongji Medical College, Huazhong University of Science and Technology, Wuhan, China

**Keywords:** ketogenic diet, tumor, meta-analysis, survival time, animal studies

## Abstract

**Background:** The ketogenic diet (KD) has been reported to play an important role in the development of cancer by an abundance of pre-clinical experiments; however, their conclusions have been controversial. We therefore aimed to perform a systematic review and meta-analysis of animal studies evaluating the effects of KD on cancer.

**Methods:** Relevant studies were collected by searching PubMed, Embase, and Web of Science. Outcome measures comprised tumor weight, tumor volume, and survival time. Meta-analysis was performed using the random-effect model according to heterogeneity.

**Results:** The search resulted in 1,254 references, of which 38 were included in the review and 17 included in the meta-analysis. Pooled results indicated that KD supplementation significantly prolonged survival time [standardized mean difference (SMD) = 1.76, 95% CI (0.58, 2.94), *p* = 0.003], and reduced tumor weight [SMD = −2.459, 95% CI (−4.188, −0.730), *p* = 0.027] and tumor volume [SMD = −0.759, 95% CI (−1.349, −0.168), *p* = 0.012]. Meta-regression and subgroup analysis results suggested that KD supplementation at a ratio of 4:1 was associated with remarkable prolongation of survival time in animals with limited tumor types.

**Conclusion:** In summary, the pre-clinical evidence pointed toward an overall anti-tumor effect of the KD in animals studies currently available with limited tumor types.

## Introduction

Cancer is one of the major problems worldwide and is grievously harmful to human health (1). Recently, it has been found that tumor metabolic reprogramming is a central feature of tumors (2). The Warburg effect, as the core of tumor metabolic reprogramming, indicates that tumor cells tend to undergo aerobic glycolysis to metabolize glucose (3). Thus, reducing glucose supply and selectively cutting off the energy source of tumor cells could inhibit tumor growth (4). The ketogenic diet (KD), characterized by a high-fat, low-carbohydrate, and adequate-protein diet, can meet such demand. Therefore, ketogenic therapy for cancer has emerged and become an area of wide discussion in tumor research in recent years.

A great number of pre-clinical studies have suggested that KD is a potent anticancer therapy when used separately or as an adjuvant (5). It has been reported that KD not only slowed tumor growth and delayed the initiation of tumor development, but also prolonged survival time (6, 7). In addition, some studies have demonstrated that KD could increase the sensitivity of tumor cells to classic chemotherapy and radiotherapy when used in combination (8–10). Furthermore, KD has been reported to enhance the efficacy of targeted therapy and overcome drug resistance in several tumor models when using PI_3_K inhibitors (11), as well as reduce metastatic potential (12, 13). On the contrary, pro-tumor effects or severe side effects have been found in certain cancer models. For instance, such effects have been described in a rat model of tuberous sclerosis complex when investigating the long-term KD treatment effects on kidney cancer (14), while another study observed that tumor growth has significantly increased with KD supplementation in a mouse model of BRAF V600E-positive melanoma (15). Therefore, it is controversial whether KD has shown anti-tumor effects in pre-clinical studies.

To date, clinical evidence from randomized controlled clinical trials is still lacking, and available evidence is mostly from case reports and pilot/feasibility studies. To better understand the anti-tumor effects of KD and to pave the way for further prospective clinical studies, we performed a systematic review and meta-analysis of current available data on animal tumor models treated with KD alone or in combination with classic therapy and/or caloric restriction.

## Materials and Methods

### Literature Search

A comprehensive, computerized literature search was performed in PubMed, Embase, and Web of Science up to April, 2020 using the following key words: “ketogenic,” “caloric restriction” paired with the following: “glioma,” “glioblastoma,” “tumor,” “cancer,” “neuroblastoma,” “carcinoma” (see Supplementary Table 1). References of the identified publications were then reviewed to further identify potentially relevant articles.

### Study Selection and Inclusion Criteria

Studies were included in our article if the following criteria were met: (1) published as full-length articles in English; (2) reported as animal studies; (3) the exposure of interest was KD alone or in combination with classic therapy and/or caloric restriction; and (4) reported data on at least one of the following: survival time, tumor volume, or tumor weight. The following additional exclusion criteria were used for full-text screening: (1) full text not available, (2) double publication, (3) conference abstracts, (4) review, (5) editorials, and (6) comments.

### Data Extraction and Quality Assessment

Literature search, data extraction, and quality assessment were completed independently by two authors (J.L. and H.Y.Z.) according to the inclusion criteria. In cases of disagreement between the authors, consensus was reached. The following information were extracted: the first author's name, published year, tumor type, animal species, cell strain, the ketogenic ratio, the composition of KD, whether KD was accompanied with caloric restriction, study groups, animal number of each group, survival time, tumor weight, tumor volume, the levels of glucose and β-hydroxybutyrate, the changes of body weight and conclusion. Outcome measures, including tumor weight, tumor volume, and survival time were included in the meta-analysis. The mean value, standard deviation (SD), and number of animals per group were extracted. For studies with multiple intervention groups [e.g., KD and KD + chemotherapy (CT)], the shared control group was split into 2 or more groups of smaller sample sizes to overcome unit-of-analysis errors, and these multiple comparisons were included into the meta-analysis according to instructions of the Cochrane's Handbook.

### Data Synthesis and Statistical Analysis

Given that various measurements have been applied in the included studies, the pooled effects are presented as standardized mean difference (SMD) with 95% confidence intervals (CI). The Cochrane's *Q*-test was performed to assess inter-study heterogeneity, and significant heterogeneity was considered when *p*-value was < 0.10. The *I*^2^ statistic was also examined, and an *I*^2^ value of > 50% indicated significant heterogeneity among the studies. A random effects model or fixed effects model was used according to the heterogeneity.

To explore the potential causes of heterogeneity, meta-regression analysis and pre-defined subgroup analysis were performed. Furthermore, potential publication bias was assessed using the Egger regression asymmetry test and funnel plots. All meta-analyses and statistical analyses were performed using the Stata software (version 12.0; Stata Corporation, College Station, TX, USA).

## Results

### Description of the Included Studies

**Figure 1:**
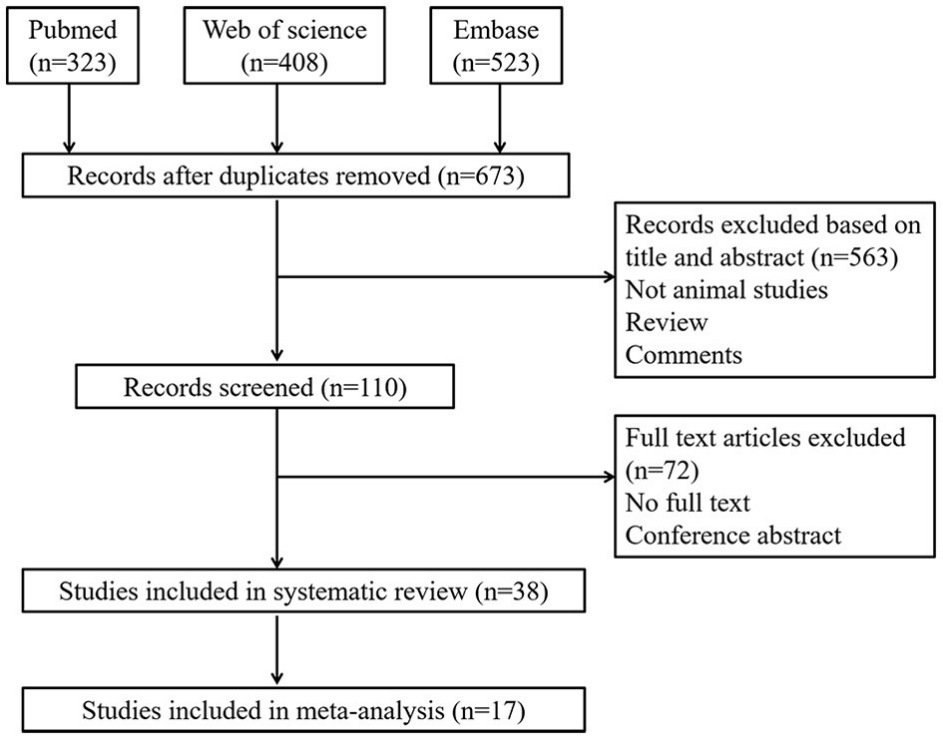
Flow diagram showing literature search and selection results.

The comprehensive search strategy on the effects of KD on tumors resulted in 1,254 records. After removal of duplicates, 673 studies remained. After title and abstract screening, the full texts of 110 studies were screened. Ultimately, 38 studies were included in our systematic review, of which 17 studies were included in the meta-analysis (Figure 1).

The characteristics of all included studies are described in Table 1 and the detailed composition of KD involved in meta-analysis are listed in Supplementary Table 2. Different mouse cancer models have been used to evaluate the anti-tumor effects of KD, including glioblastoma, breast cancer, lung cancer, melanoma, liver cancer, colon cancer, prostate cancer, pancreatic cancer, kidney cancer, neuroblastoma, anaplastic thyroid cancer, medulloblastoma, gastric cancer, and systemic metastatic cancer. The animal species included C57BL/6, BALB/c, VM/Dk, nu/nu, NMR1, CDF1, SCID, athymic nude mice, CD-1 nude mice, Ptch1^+/−^Trp53^−/−^ mice, Eker (Tsc2^+/−^) rats and so on. On the other hand, many cell strains were involved, including U87-MG, GL261-Luc2, VM-M3, 4T1, ES272, NCI-H292, A549, LLC1, SH-SY5Y, 786-O, 8505C, LNCaP LAPC-4, et al. KD ratios ranging from 1:1 to 8:1 were widely used across studies. Although some studies reported pro-tumor effects or no effects, most studies suggested that dietary interventions with KD constituted potent anti-cancer therapy. Besides, as a monotherapy, KD has also been used as an adjuvant for chemotherapy, radiotherapy or hyperbaric oxygen therapy, or in combination with metformin.

**Table 1:** Animal studies reporting the effect of the KD on tumor growth and survival.

**Tumor**	**References**	**Animal species**	**Cell strain**	**KD ratio**	**CR**	**Study groups**	**Animal number**	**Tumor growth**	**Survival time**	**G, β-HB and BW**	**Conclusion**
Glioblastoma	Zhou et al. (16)	C57BL/6J; BALBc/J-SCID mice	U87-MG	4:1	Yes	SD; KD; KD-CR	*n* = 12–14	KD vs. SD: ns	KD vs. SD: ns	KD-CR reduces G and BW; KD-CR elevates β-HB.	KD-CR has anti-tumor effect.
								KD-CR vs. SD: inhibition	KD-CR vs. SD: increase		
	Stafford et al. (17)	C57BL/6 mice	GL261	8:1	No	SD; KD	*n* = 20	–	KD vs. SD: increase	G: ns; β-HB: increase; BW: ns.	KD improves survivability
	Abdelwahab et al. (8)	Albino C57BL/6 mice	GL261-Luc2	4:1	No	SD; KD; SD + RT; KD + RT	*n* = 11–19	Inhibition	KD vs. SD: increase; KD + RT vs. SD + RT: no data	G: decrease; β-HB: increase; BW: reduce.	KC enhances survival and slows tumor growth; additive effect of KD + RT
	Rieger et al. (18)	Athymic nude mice	U87MG	3.14:1	No	SD; KD; SD + CT; KD + CT	*n* = 5–7	KD vs. SD: ns; KD + CT vs. SD + CT: inhibition	KD + CT vs. SD + CT: increase	G: ns; β-HB: increase; BW: ns.	No effect of KD alone; enhanced survival of KD + CT vs. SD + CT
	Woolf et al. (19)	Albino C57BL/6 mice	GL261-Luc2	4:1	No	SD; KD	*n* = 6	–	–	G: decrease; β-HB: increase; BW: reduce.	Alters the hypoxic
											Response, reduced tumor microvasculature
	Lussier et al. (7)	Albino C57BL/6 mice	GL261-Luc2	4:1	No	SD; KD	*n* = 12	–	KD vs. SD: increase	G: decrease; β-HB: increase; BW: ns.	KD enhances survival
	De Feyter et al. (20)	Fischer rats	RG2, 9L	4:1	Yes	SD-CR; KD-CR	*n* = 10–11	ns	ns	G: decrease; β-HB: increase; BW: reduce.	No effect
	Augur et al. (21)	VM/Dk mice	VM-M3	2.7:1	No	SD; KD	*n* = 4–9	–	ns	G: decrease; β-HB: increase; BW: reduce.	No effect
	Mukherjee et al. (22)	VM/Dk mice	VM-M3	4:1	Yes	SD; KD-CR; SD + DON; KD-CR + DON	*n* = 10–15	–	Increase	CR-KD ± DON decreases G and increases β-HB; BW: reduce.	KD-CR + DON prolong survival time
Breast	Gluschnaider (23)	Transgenic FVB MMTV-PyMT mice	Spontaneous tumor development	4:1	No	SD; HFD; KD; MEDICA	*n* = 8–10	KD vs. SD: inhibition	–	BW: ns.	Anti-tumor
	Zhuang et al. (24)	BALB/c mice	4T1	6:1	Yes	SD; SD + Metformin; KD-CR; KD-CR + Metformin	*n* = 10	Inhibition	–	G: decrease	KD-CR have anti-tumor effects; additive effect of KD + Metformin
	Hopkins et al. (11)	C57BL/6 mice	ES272	6:1	No	SD; SD + PI_3_K inhibitors; KD; KD + PI_3_K inhibitors	*n* = 5	Inhibition	–	–	No effect of KD alone, additive effect of KD + PI3K inhibitors
Lung	Allen et al. (9)	nu/nu mice	NCI-H292, A549	4:1	No	SD; KD; IR; IR + KD; CT; CT + KD; IR + CT; IR + CT + KD	*n* = 5–16	Inhibition	KD + RT: prolongs survival; KD + RT/CT: prolongs survival	β-HB: increases	No effect of KD alone, enhanced anti-tumor effects of KD + CT/RT
	Stemmer et al. (25)	C57BL/6 (Fgf21 WT and KO) mice	LLC1	3:1; 8:1	No	SD; RP-KD; LP-KD	*n* = 8–15	Inhibition	–	Low protein KD decreases G and increases β-HB. BW: ns.	LP-KD has anti-tumor effect
Liver	Healy et al. (26)	C57BL/6N mice	DEN-induced hepatocellular carcinoma	5:1	No	SD; WD-L; WD-C; FD; KD	*n* = 6–12	–	–	G: ns; BW: ns.	Anti-tumor
	Byrne et al. (27)	C57BL/6N mice	DEN-induced hepatocellular carcinoma	4:1	No	SD; KD	*n* = 7	ns	-	β-HB: increase; BW: increase.	No effect
BRAF V600E-positive A375 melanoma	Xia et al. (15)	nu/nu mice	A375, A2058 (BRAF V600E)	4:1; 6:1	No	SD; KD	*n* = 8	Increase	–	G: decreases; β-HB: ns; BW: ns.	Pro-tumor
SKMEL-2 cells melanoma	Xia et al. (15)	nu/nu mice	SK-MEL-2 (NRAS Q61R)	4:1	No	SD; KD	*n* = 7	ns	-	G: decreases; β-HB: ns; BW: ns.	No effect
Colon	Tisdale et al. (28)	NMR1 mice	MAC16	1:1; 2:1	No	SD; 68%fat KD ± 3-hydroxybutyrate; 80% fat KD ± 3-hydroxybutyrate	ns	80% fat KD ± 3-hydroxybutyrate decreases tumor weight	-	G: ns; β-HB: increase; BW: reduce.	Anti-tumor
	Beck and Tisdale (29)	NMR1 mice	MAC16	2:1	No	SD; KD	*n* = 6–12	KD decreases tumor weight	–	G: ns; β-HB: increase; BW: reduce.	Anti-tumor
	Hao et al. (30)	BALB/c nude mice	HCT-116	3:1	No	SD; MKD; LKD	*n* = 12	KD inhibits tumor growth	KD prolongs survival time	G: ns; β-HB: increase; BW: increase.	Anti-tumor
	Nakamura et al. (31)	CDF1 mice	Colon 26	3:1	No	SD; KD	*n* = 5–10	KD decrease tumor weight	–	β-HB: increase; BW: reduce.	Anti-tumor
	Kasumi and Sato (32)	BALB/c mice	Colon 26	4:1	No	SD; KD	*n* = 6–8	ns	KD prolongs survival time	G: ns; β-HB: increase; BW: reduce.	KD improves the prognosis
Prostate	Freedland et al. (33)	SCID mice	LAPC-4	2:1	No	Low fat; Western diet; no-carbohydrate ketogenic diet (NCKD)	*n* = 11	ns	NCKD prolong survival time	G: increase; β-HB: increase; BW: increase.	Anti-tumor
	Mavropoulos et al. (34)	Fox chase SCID mice	LNCaP	2:1	No	NCKD; LFD; MCD	*n* = 11	NCKD decreases tumor volume	NCKD prolongs survival time	G: ns; β-HB: increase; BW: decrease.	Anti-tumor
	Kim et al. (35)	Athymic nude mice	LAPC-4	2:1	No	SD ± MCT1 inhibitor; KD ± MCT1 inhibitor	ns	KD decreases tumor volume	KD prolongs survival time	G: ns; BW: ns.	Anti-tumor
Pancreatic cancer	Shukla et al. (36)	Athymic nude mice	S2-013	2:1	No	SD; KD	*n* = 9	KD decreases tumor weight and tumor volume	–	G: decrease; β-HB: increase; BW: reduce.	Anti-tumor
	Zahra et al. (10)	Athymic nude mice	MIA PaCa-2	4:1	No	SD; KD; RT; KD + RT	*n* = 9–16	KD + RT decreases tumor volume	KD + RT prolongs survival time	G: decrease; β-HB: increase; BW: reduce.	No effect of KD alone, additive anti-tumor effect of KD + CT
	Zhang et al. (37)	nu/nu mice	PANC-1	3:1	No	SD; KD	*n* = 8	KD decreases tumor weight	KD prolong survival time	G: decrease; β-HB: increase; BW: reduce.	Anti-tumor
Kidney cancer	Liskiewicz et al. (14)	Eker (Tsc2^+^/^−^) rats	Spontaneous tumor development	8:1	No	SD; KD	*n* = 1–34	KD increases renal tumor growth	–	G: decrease; β-HB: increase	Pro-tumor
	Vidali et al. (38)	CD-1 nude mice	786-O	8:1	No	SD; LCT-KD; MCT-KD	*n* = 5–6	KD tends to slow down tumor growth, but with no significance.	KD reduces the overall survival	LCT-KD increases β-HB; BW: reduce.	KD might be contraindicated in the treatment of RCC patients presenting with Stauffer's syndrome
Anaplastic thyroid cancer	Aggarwal et al. (39)	CD-1 nude mice	8505C		No	SD; KD; SD + N AC; KD + NAC	*n* = 6	KD or KD + NAC	–	G: decrease; β-HB: increase	Anti-tumor
Neuroblastoma	Morscher et al. (40)	CD-1 nude mice	SH-SY5Y (non-NMYC amplified); SK-N-BE(2) (NMYC amplified)	2:1	Yes	SD; CR-SD; KD; CR-KD	*n* = 8–11	CR-SD, CR-KD decrease tumor volume	CR-SD, CR-KD prolong survival time	CR-KD decreases G and BW; CR-KD increases β-HB.	No effect of KD alone; enhanced effects of CR-KD
	Morscher et al. (41)	CD-1 nude mice	SH-SY5Y (non-NMYC amplified);	2:1	Yes	Cell line: SH-SY5Y (non-NMYC amplified)	*n* = 8–12	KD + CT, CR-KD + CT decrease tumor volume	KD + CT, CR-KD + CT prolong survival time	CR-KD decreases G and increases β-HB; BW: reduce.	Antitumor effects of KD + CT; additive effect of CR on KD + CT
						SD; SD + CT; CR-SD + CT; KD + CT; KD-CR + CT					
	Morscher et al. (41)	CD-1 nude mice	SK-N-BE(2) (NMYC amplified)	2:1	Yes	Cell line: SK-N-BE(2) (NMYC amplified)	*n* = 8–12	KD + CT: ns; CR-KD + CT decreases tumor volume	KD + CT: ns; CR-KD + CT prolongs survival time	CR-KD decreases G and increases β-HB; BW: reduce.	No effect of KD + CT; enhanced anti-tumor of CR on KD + CT
						SD; SD + CT; CR-SD + CT; KD + CT; KD-CR + CT					
	Aminzadeh-Gohari et al. (42)	CD-1 nude mice	SH-SY5Y (non-NMYC amplified); SK-N-BE(2) (NMYC amplified)	8:1	No	SD; LCT-KD; MCT-KD	*n* = 10–12	LCT-KD and MCT-KD decrease tumor volume	MCT-KD prolongs survival time	G: decrease; β-HB: increase; BW: ns.	Anti-tumor
Medulloblastoma	Dang et al. (43)	Ptch1^+^/^−^Trp53^−^/^−^mice	Spontaneous tumor development	4:1	No	SD; KD	*n* = 4	ns	ns	BW: reduce.	No effect
	Dang et al. (43)	NOD SCID mice	Medulloblastoma from Ptch1^+^/^−^Trp53^−^/^−^ mice	6:1	No	SD; KD	*n* = 4	ns	ns	G: decrease; β-HB: increase; BW: reduce.	No effect
Gastric cancer	Otto et al. (6)	NMRI nude mice	23132/87	2.7:1	No	SD; KD	*n* = 12	KD inhibits tumor growth	KD prolongs survival time	G: ns; β-HB: increase; BW: ns.	Anti-tumor
Systemic metastatic cancer	Poff et al. (12)	VM/Dk mice	VM-M3	4:1	No	SD; SD + HBO_2_T; KD; KD + HBO_2_T	*n* = 8–13	KD, KD + HBO_2_T inhibit tumor growth	KD, KD + HBO_2_T prolong survival time	G: decrease; β-HB: ns; BW: reduce.	Anti-tumor; enhanced antitumor effects of HBO_2_T on KD
	Poff et al. (13)	VM/Dk mice	VM-M3	1.5:1	No	SD; KD; KD + KE:KD + KE + HBO_2_T	*n* = 7–17	KD, KD + KE, KD + KE + HBO_2_T inhibit tumor growth	KD, KD + KE, KD + KE + HBO_2_T prolong survival time	KD + KE, KD + KE + HBO_2_T decrease G and increase β-HB; BW: reduce.	Anti-tumor

*KD, ketogenic diet; CR, calorie-restricted; RT, radiotherapy; CT, chemotherapy; DON, 6-diazo-5-oxo-L-norleucine; RP, regular protein; LP, low- protein; WD-L, Western diet based on lard; WD-C, Western diet based on coconut oil; FD, fructose diet; NCKD, high-fat/no-carbohydrate ketogenic diet; LFD, low-fat/high-carbohydrate diet; MCD, high-fat/moderate-carbohydrate diet; NAC, N-acetylcysteine; HBO_2_T, hyperbaric oxygen therapy; KE, ketone ester; G, glucose; β-HB, b-hydroxybutyrate; BW, body weight*.

### Effects of KD on Survival Time in Animal Models

**Figure 2:**
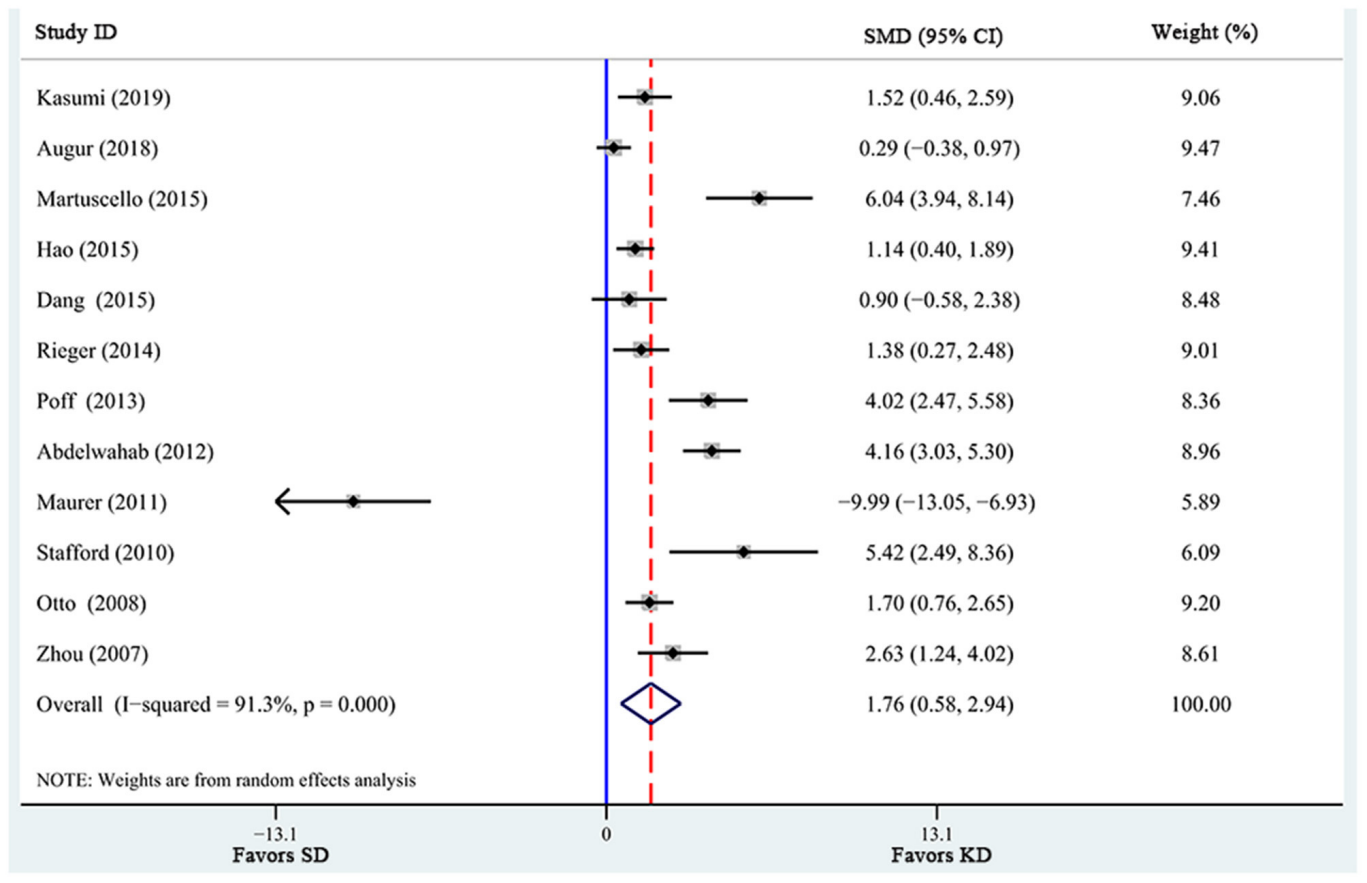
Forest plot from meta-analysis of standardized mean difference in survival time of animal tumor models randomized to ketogenic diet (KD) or standard diet (SD). The effect size of each study is proportional to the statistical weight. The diamond indicates the overall summary estimate for the analysis; the width of the diamond represents the 95% CI. SMD, standardized mean difference; CI, confidence interval.

There were a total of 12 studies investigating the effects of KD supplementation on survival time (Table 2). Significant heterogeneity was found among these studies (*I*^2^ = 91.3%, *p* = 0.000). Pooled analysis of the overall effects suggested that KD significantly prolonged survival time [SMD = 1.76, 95% CI (0.58, 2.94), *p* = 0.003; Figure 2] in animal models.

**Table 2:** Studies fulfilling all inclusion criteria for the meta-analysis on survival time: outcome data.

**References**	**KD ratio**	**SD group**		**KD group**	
		***n***	**Mean ± SD**	***n***	**Mean ± SD**
Kasumi and Sato (32)	4	9	19.8, 1.7	9	23.7, 3.2
Augur et al. (21)	3	17	15.2, 2.6	17	16.1, 3.5
Martuscello et al. (44)	6	11	38, 1	10	56, 4.2
Hao et al. (30)	3	12	24.8, 3.1	24	34.51, 0.1
Dang et al. (43)	4	4	16.3, 2.3	4	17.8, 0.5
Rieger et al. (18)	3	8	33.9, 1.6	8	35.6, 0.7
Poff et al. (12)	4	13	31.2, 4.4	8	48.9, 4.4
Abdelwahab et al. (8)	4	19	23.3, 1.1	20	28.8, 1.5
Maurer et al. (45)	3	12	94.9, 1.3	12	24, 1.2
Stafford et al. (17)	6	5	19, 0.7	5	34.2, 1.1
Otto et al. (6)	3	12	23.3, 3.1	12	19.7, 8.5
Zhou et al. (16)	4	7	16.7, 1.4	9	23.7, 0.9

### Meta-Regression Analysis and Subgroup Analysis

In view of the fact that statistical heterogeneity existed across the included studies, meta-regression analysis was performed by including several pre-defined covariates to explore the potential sources of heterogeneity. The results indicated that KD ratio was positively related to effect size [regression coefficient = 1.69, 95% CI (0.86, 2.52), *p* = 0.02]. Furthermore, animal number was not a significant modifier to the effects of KD supplementation on survival time (*p* = 0.655).

**Figure 3:**
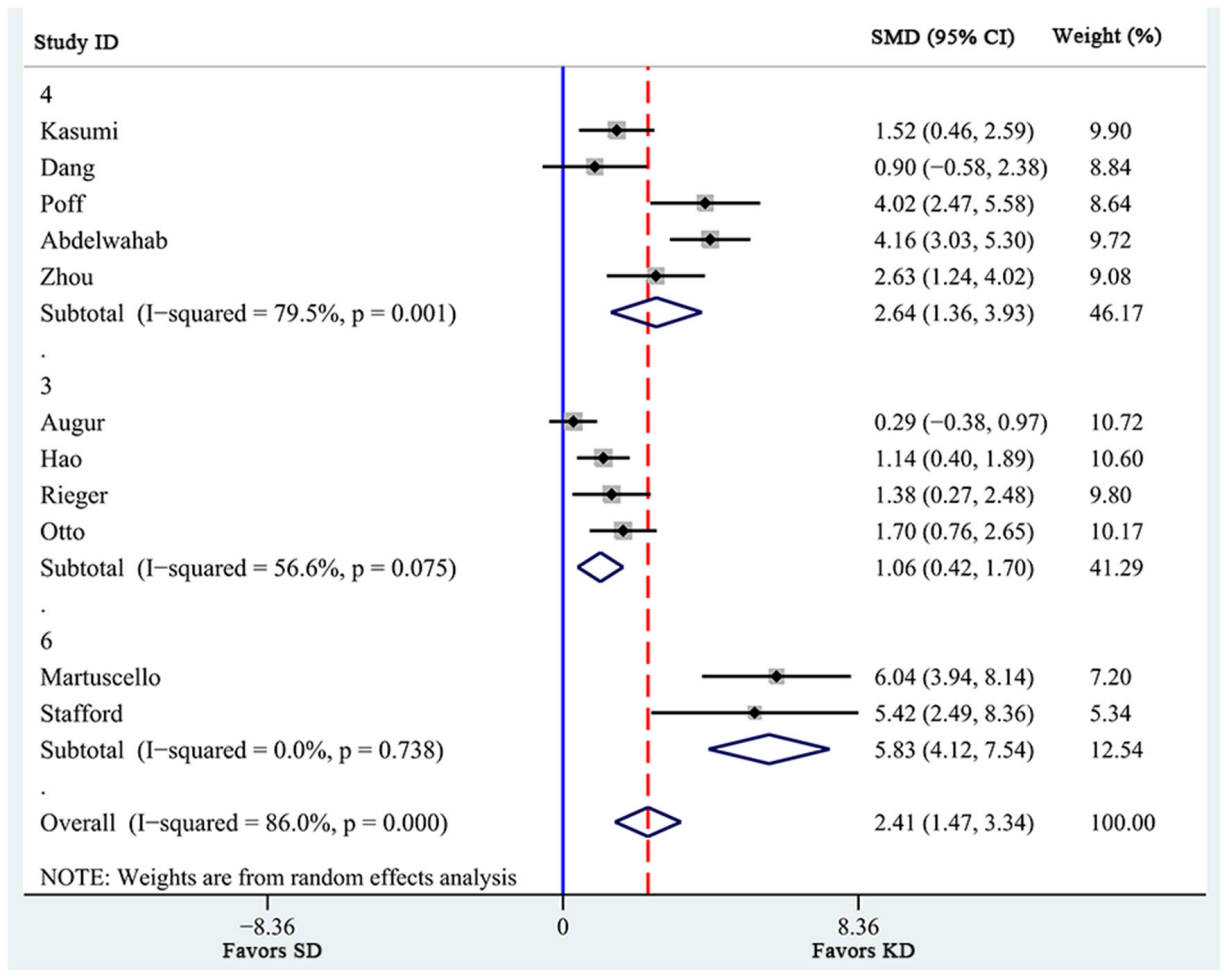
Subgroup estimation of the effects of ketogenic diet supplementation on survival time. The numbers 3, 4, and 6 represent the KD ratio. The effect size of each study is proportional to the statistical weight. The diamond indicates the overall summary estimate for the analysis; the width of the diamond represents the 95% CI. SMD, standardized mean difference; CI, confidence interval.

Additionally, a pre-defined subgroup analysis was conducted to observe the influence of study characteristics on the effects of KD supplementation on survival time. First, 3 subgroups were obtained according to KD ratio. As shown in Figure 3, even though all 3 subgroups showed significantly prolonged survival time, the effects of KD ratios of 4 [SMD 2.64 (1.36, 3.93), *n* = 5] seemed to be larger than those of 3 [SMD 1.06 (0.42, 1.70), *n* = 3]. In addition, heterogeneity levels significantly decreased in the subgroup analysis of KD ratios of 6 (*I*^2^ = 0.0%), while high heterogeneity levels were still observed in subgroups with ratios of 4 (*I*^2^ = 79.5%) and of 3 (*I*^2^ = 56.6%). Specifically, KD supplementation with a ratio of 4 seemed to be associated with more remarkable prolongation of survival time in animals (*p* = 0.001).

### Effects of KD on Tumor Weight and Tumor Volume

**Figure 4:**
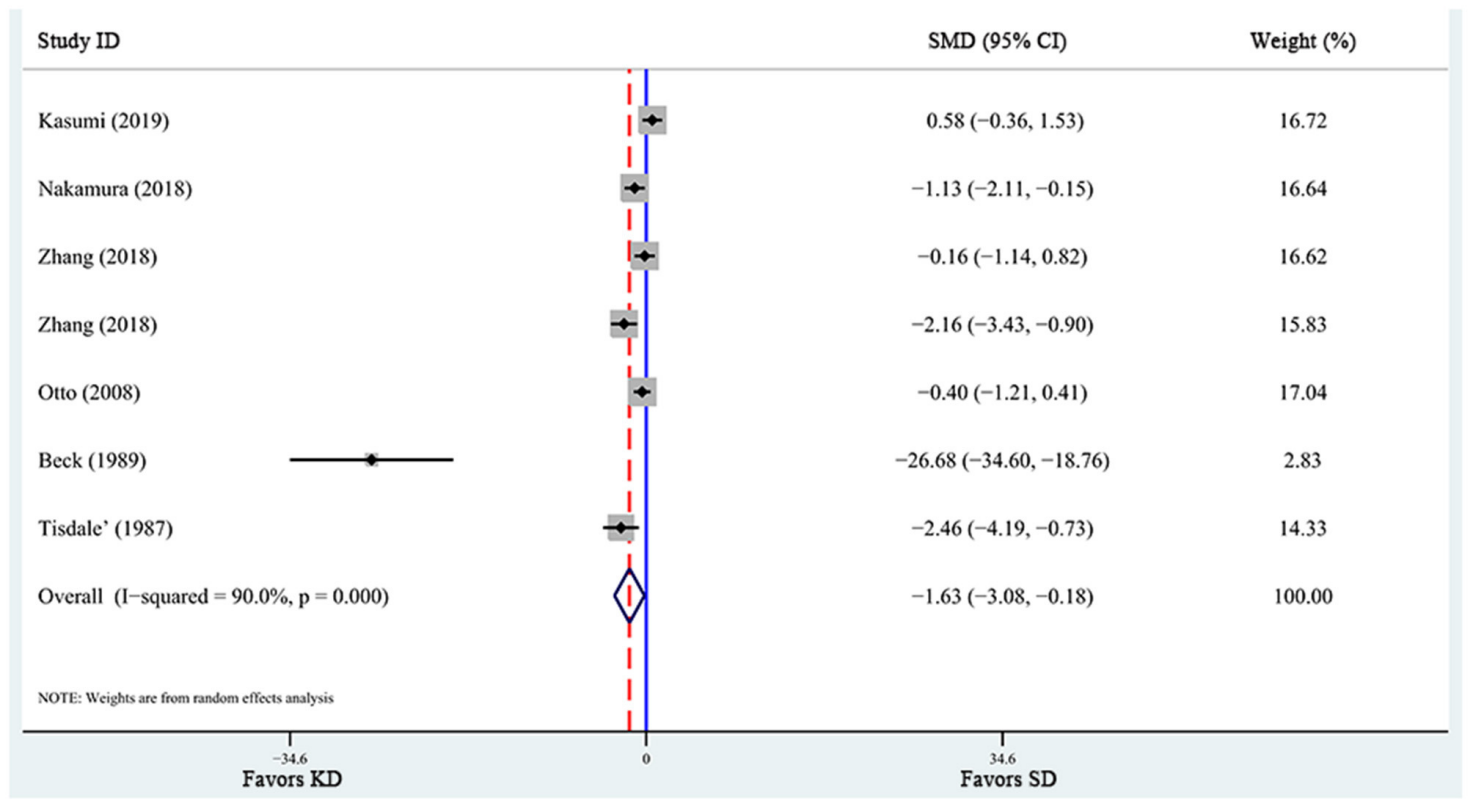
Forest plot from meta-analysis of standardized mean difference in tumor weight of animal tumor models randomized to ketogenic diet (KD) or standard diet (SD). The effect size of each study is proportional to the statistical weight. The diamond indicates the overall summary estimate for the analysis; the width of the diamond represents the 95% CI. SMD, standardized mean difference; CI, confidence interval.

**Figure 5:**
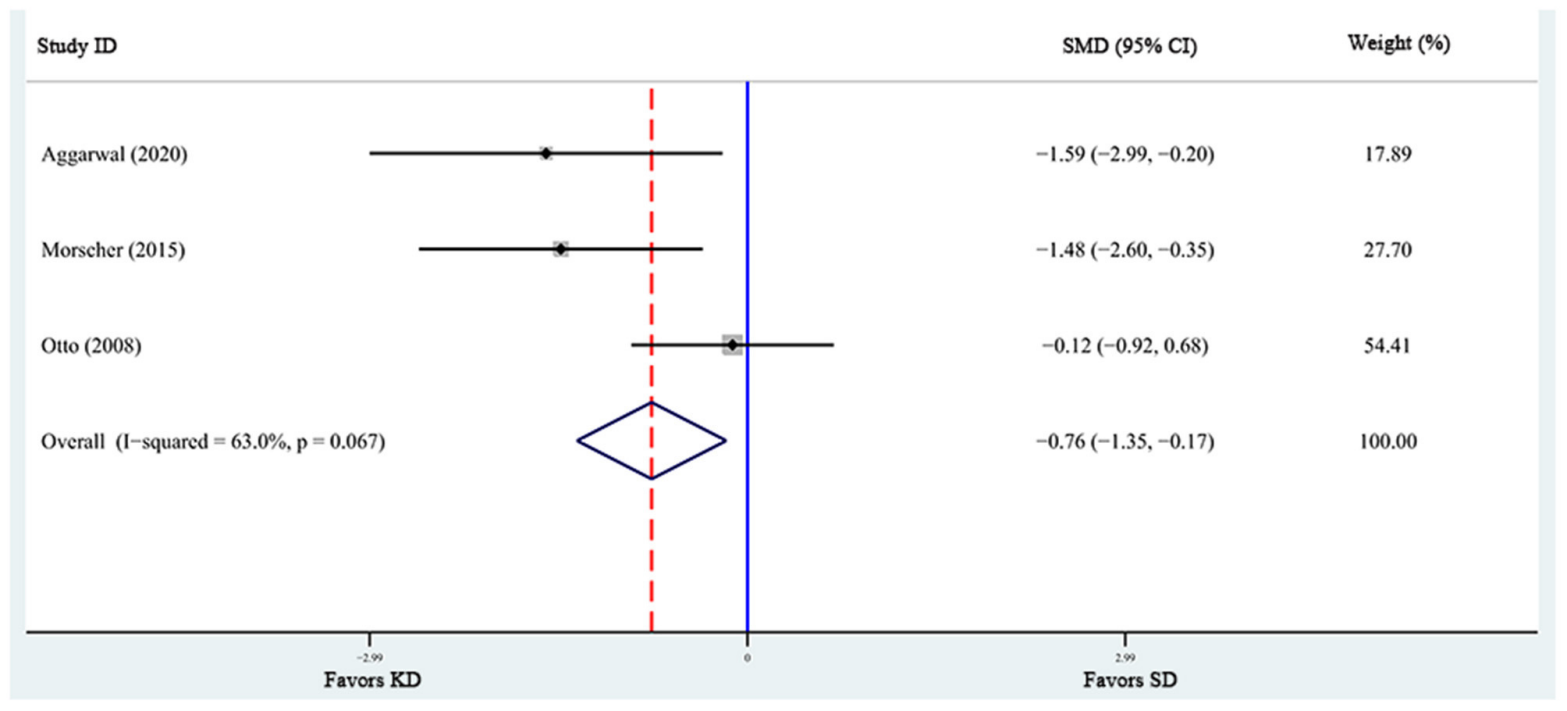
Forest plot from meta-analysis of standardized mean difference in tumor volume of animal tumor models randomized to ketogenic diet (KD) or standard diet (SD). The effect size of each study is proportional to the statistical weight. The diamond indicates the overall summary estimate for the analysis; the width of the diamond represents the 95% CI. SMD, standardized mean difference; CI, confidence interval.

A total of 6 articles (6, 28, 29, 31, 32, 37), including 7 studies, reported the effects of KD supplementation on tumor weight in animal models. The overall effects were estimated using a random-effect model because significant heterogeneity was found (*I*^2^ = 90%, *p* = 0.000). Meanwhile, significant heterogeneity also existed in tumor volume (6, 39, 40) (*I*^2^ = 63%, *p* = 0.067). The pooled results indicated that KD significantly reduced tumor weight and tumor volume [tumor weight: SMD = −2.459, 95% CI (−4.188, −0.730), *p* = 0.027, Figure 4; tumor volume: SMD = −0.759, 95% CI (−1.349, −0.168), *p* = 0.012, Figure 5]. Meta-regression and subgroup analyses were not performed for these outcomes because of the limited number of studies included.

### Publication Bias

**Figure 6:**
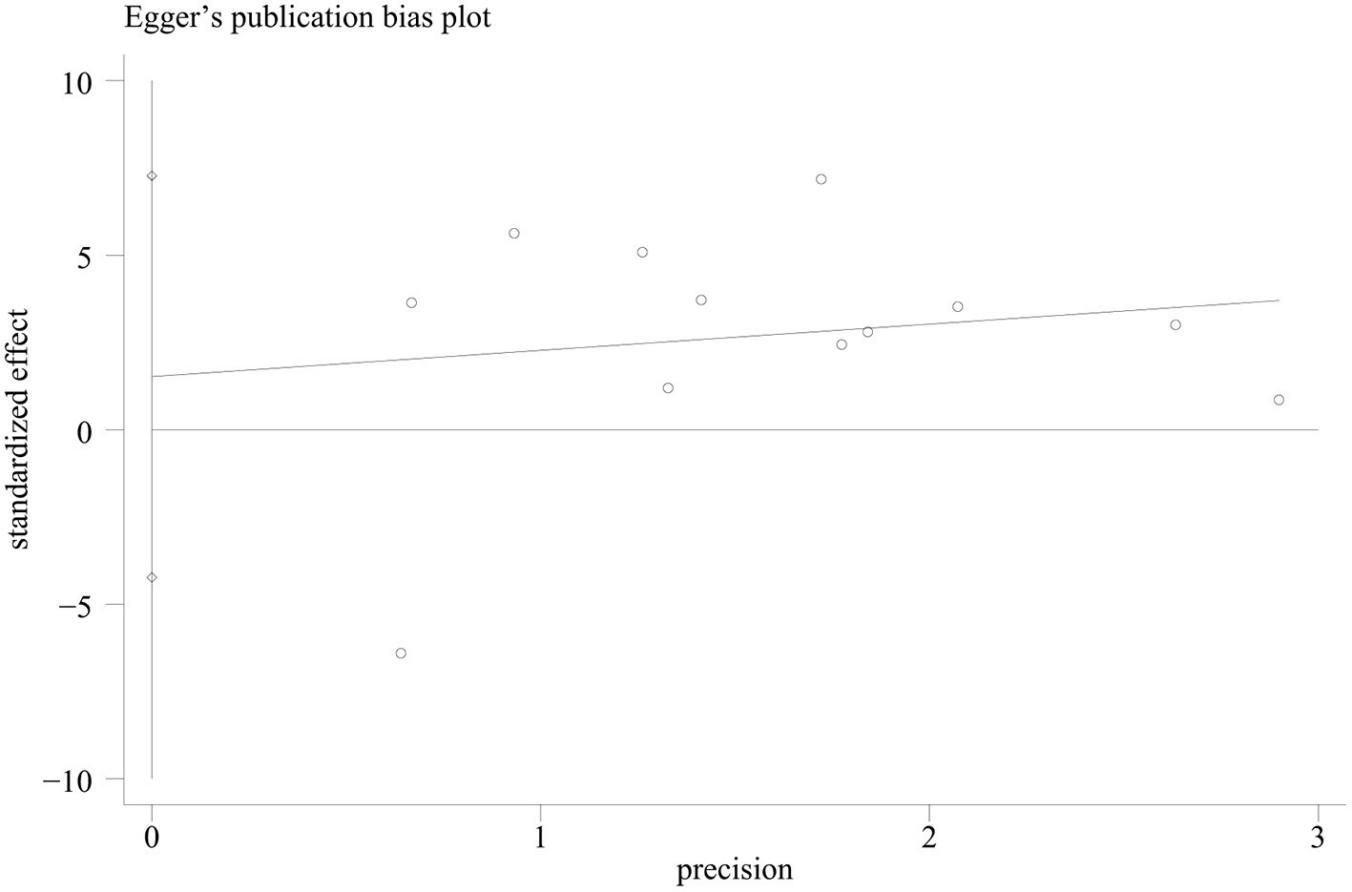
The Egger's test regression plot in the meta-analysis of survival time of animal tumor models randomized to ketogenic diet (KD) or standard diet (SD).

Publication bias was assessed for the outcome of overall survival time, since this outcome has been analyzed in the highest number of studies. The Egger regression asymmetry test of the 12 studies suggested no significant publication bias for survival time [*p* = 0.569, 95% CI (−4.23, 7.27), Figure 6].

## Discussion

In this meta-analysis, we summarized evidence from 17 published animal studies that investigated the effects of KD supplementation on anti-tumor effects. Consistent with the previous meta-analysis which reported that unrestricted KD delayed tumor growth in mice (46), our results showed that KD alone or in combination with caloric restriction significantly reduced tumor weight and volume as well as prolonged survival time. Results of our meta-regression and subgroup analyses suggested that KD supplementation with a ratio of 4 seemed to associate with remarkable prolongation of survival time in animals with limited tumor types.

Traditional KD consisted of a 4:1 ratio of fat to carbohydrate plus protein, with calories from fat, protein, and carbohydrate being 90, 8, and 2%, respectively. Other alternatives to traditional KD include the medium-chain triglyceride (MCT)-based KD, the Atkins diet, and a low glycemic index diet (47). In order to enhance the anti-tumor effects of KD, several studies have either increased the proportion of fat, or supplemented KD with MCTs, omega-3 fatty acids or ketone esters (6, 13, 30, 42, 44). For example, Aminzadeh-Gohari et al. found that KD (8:1) with a fat content of 25% MCTs and 75% long-chain triglycerides (LCTs) produced a stronger anti-tumor effect compared to that with only LCTs (42). The reason may be that MCTs are more rapidly absorbed into the bloodstream and oxidized for energy because of their ability to passively diffuse through membranes (48). In addition, MCTs have the unique ability to promote ketone body synthesis in the liver (49). Tisdale et al.'s study indicated that high fat KD (2:1) showed a significant reduction in tumor size when compared with normal diet and low fat KD (1:1) (28). These results demonstrated that it is important to optimize KD compositions to suppress tumor growth.

Totally, KDs have revealed the potential anti-tumor effects, which is correlated with the restricted glucose and induction ketone bodies (e.g., β-hydroxybutyrate) (50). Ketone bodies are suitable energy replacements for normal cells with functional mitochondria, but unsuitable for tumor cells, as tumor cell mitochondrial functions are dysregulated (51). Indeed, most animal tumor models report decrease of glucose and increase of ketone bodies (Table 1). On the other hand, KDs are known to have an appetite suppressing effect which may contribute to body weight loss (52), while some studies report no significant effect or increase of body weight. The discrepancy may be caused by the animal species and growth stage, or the composition the KD.

Caloric restriction (CR) has been reported to prevent tumorigenesis by decreasing metabolic rate and oxidative damage (53). Morscher et al. found that the growth of neuroblastoma xenografts was significantly reduced by KD (2:1) when combined with CR (40). Another study indicated that anti-tumor and anti-angiogenic effects were revealed in experimental mouse and human brain tumors at a 4:1 KD ratio (16). It is reported that tumor growth is more strongly correlated with circulating glucose levels than with circulating ketone body levels (51). The reduction in glucose levels following CR largely accounts for why tumors grow minimally on either restricted KD or on restricted high carbohydrate standard diets. Although CR exhibited good anti-tumor effects and the potential to sensitize cancer cells to chemotherapy, CR has been considered to be contraindicated in a range of cancer patients, particularly those with cachexia (5). Thus, more attention is required on optimizing KD compositions to enhance the anti-tumor effects.

The efficacy of KD may also be influenced by cancer type or even subtype, genetic background, and tumor-associated syndromes. KD with a ratio of 4:1 did not slow the growth of spontaneous medulloblastoma tumors or allograft flank tumors (43), while it was reported to be anti-tumor in other cancer models, including glioblastoma (7) and colon cancer (32). Meanwhile, one study indicated that the anti-neuroblastoma effects of KD were considerably attenuated in SKN-BE(2) neuroblastoma xenografts, which carried MYCN amplification, TP53 mutation (p.C135F), and chromosome 1p loss of heterozygosity, compared to SH-SY5Y xenografts which are TP53 wild-type and non-MYCN amplified (42). Another report indicated that mice bearing renal cell carcinoma xenografts with signs of Stauffer's syndrome experienced dramatic weight loss and liver dysfunction when treated with KD (38). Additionally, Maurer et al. found that a KD did not alter tumor growth or extend the life of mice given an orthotopic injection of LNT-229 glioma cells when compared to mice maintained on SD (45). This is in contrast to the study using a rodent KD (17). This discrepancy may be related, in part, to the cell line and/or model system used. Therefore, it is necessary to evaluate the effects of KD in pre-clinical studies for every specific type of tumor before its application to cancer patients. Furthermore, genetic alterations, tumor-associated syndromes, and anti-tumor mechanisms of KD should also be considered.

To date, human data on KD and cancer are mostly single case reports (54, 55) or pilot/feasibility studies (56–58), which have mostly focused on the safety and tolerability of KD. Only 3 randomized controlled trials are available. Two of them involved ovarian and endometrial cancer, and mainly focused on safety, adherence, and the mental and physical functions (59, 60). The other trial evaluated the safety, tolerability, and beneficial effects of KD on body composition, blood parameters, and survival in breast cancer (61), which suggested that chemotherapy combined with KD can improve the biochemical parameters, body composition, and overall survival with no substantial side effects in breast cancer patients. Thus, it is still necessary for more randomized controlled trials to explore the benefits of adjuvant KD in specific cancers.

Several potential limitations should be addressed in the present meta-analysis. First, we did not have complete access to every full text papers, resulting in a small number of studies included in this meta-analysis; results of some of the estimations, such as those for the effects of KD supplementation on tumor weight and tumor volume, should therefore be interpreted with caution. In addition, despite the attempts to explore the potential causative factors of heterogeneity, high heterogeneity was found among the studies. In addition, the number of included studies in the subgroup analysis was relatively small.

## Conclusion

In summary, the pre-clinical evidence pointed toward an overall anti-tumor effect of the KD in animals studies currently available with limited tumor types. The efficacy of KD on tumor influenced by many factors, including cancer type or even subtype, genetic background, cell line and/or model system, the composition of KD and tumor-associated syndromes. Therefore, more pre-clinical studies should be performed to elaborate the anti-tumor effect of KD in the future.

## Data Availability Statement

The original contributions presented in the study are included in the article/Supplementary Material, further inquiries can be directed to the corresponding author.

## Author Contributions

ZD conceived of the study idea. JL and HZ conducted the literature review and performed the data extraction. JL drafted the manuscript. All authors were involved in consensus agreements concerning data discrepancies, involved in revising the article for important intellectual content, interpreting the data, and approved the final version to be published.

## Conflict of Interest

The authors declare that the research was conducted in the absence of any commercial or financial relationships that could be construed as a potential conflict of interest.
