# Supplementary material for: Cancer Treatment With the Ketogenic Diet: A Systematic Review and Meta-analysis of Animal Studies
Source: Front Nutr. 2021 Jun 9;8:594408. doi: 10.3389/fnut.2021.594408 (PMC8219874; doi:10.3389/fnut.2021.594408)
Supplement: Supplementary file 1 [file Table_1.DOCX]

**Table S1 Search Strategy.**

| **Data source (Number of literatures)** | **Components** |
| --- | --- |
| **Pubmed:323** | **Component 1:** (ketogenic[Title/Abstract]) OR (carbohydrate restriction[Title/Abstract]) |
|  | **Component 2:** (((((tumor[Title/Abstract]) OR (cancer[Title/Abstract])) OR (carcinoma[Title/Abstract])) OR (Glioma[Title/Abstract])) OR (glioblastoma[Title/Abstract])) OR (neuroblastoma[Title/Abstract]) |
|  | **Component 3:** ((ketogenic[Title/Abstract]) OR (carbohydrate restriction[Title/Abstract])) AND ((((((tumor[Title/Abstract]) OR (cancer[Title/Abstract])) OR (carcinoma[Title/Abstract])) OR (Glioma[Title/Abstract])) OR (glioblastoma[Title/Abstract])) OR (neuroblastoma[Title/Abstract])) |
| **Web of science:621** | **Component 1:** TI=(ketogenic) OR AB=(ketogenic) OR TI=(carbohydrate restriction) OR AB=(carbohydrate restriction) |
|  | **Component 2:** TI=(Glioma) OR AB=(Glioma) OR TI=(glioblastoma) OR AB=(glioblastoma) OR TI=(tumor) OR AB=(tumor) OR TI=(cancer) OR AB=(cancer) OR TI=(neuroblastoma) OR AB=(neuroblastoma) OR TI=(carcinoma) OR AB=(carcinoma) |
|  | **Component 3:** **Component 1** AND **Component 2** |
| **Embase:523** | **Component 1:**  ketogenic:ti AND diet:ti OR (ketogenic:ab AND diet:ab) OR (carbohydrate:ti AND restriction:ti) OR (carbohydrate:ab AND restriction:ab) |
|  | **Component 2:**  glioma:ti OR glioma:ab OR glioblastoma:ti OR glioblastoma:ab OR tumor:ti OR tumor:ab OR cancer:ti OR cancer:ab OR neuroblastoma:ti OR neuroblastoma:ab OR carcinoma:ti OR carcinoma:ab |
|  | **Component 3:** **Component 1** AND **Component 2** |

**Table S2 The composition of standard and ketogenic diets.**

|  | Standard diet | Ketogenic diet |
| --- | --- | --- |
| Kasumi^[20]^ | Casein 20.3 g/100g; Soybean Oil 7.0 g/100g; Dextrin 63.2 g/100g; AIN-93G mineral mix 3.5 g/100g; AIN-93 vitamin mix 1.0 g/100g; Cellulose 5.0 g/100g. | Casein 14.4 g/100g; Soybean Oil 72.4 g/100g; Dextrin 3.7 g/100g; AIN-93G mineral mix 3.5 g/100g; AIN-93 vitamin mix 1.0 g/100g; Cellulose 5.0 g/100g. |
| Martuscello^[26]^ | Fat 115 g/kg; Carbohydrates 570 g/kg; Protein 208 g/kg; Fiber 72 g/kg. | Fat 404 g/kg; Carbohydrates 125 g/kg; Protein 355 g/kg; Fiber 73.12 g/kg. |
| Hao^[27]^ | Protein: 22.1 g/100g; Fat (MCT: 0 g/100g; Omega-3: 0 g/100g; lard: 5.28 g/100g); Carbohydrate: 52 g/100g; AN-76 mineral mixture: 2.16 g/100g; AN-76 fiber mixture: 1 g/100g; Fiber:4.12 g/100g. | MKD: Protein: 20 g/100g; Fat (MCT: 36.2 g/100g; Omega-3: 21.8 g/100g; lard: 11 g/100g); Carbohydrate: 3 g/100g; AN-76 mineral mixture: 2.5 g/100g; AN-76 fiber mixture: 1 g/100g; Fiber: 4 g/100g.  LKD: Protein: 20 g/100g; Fat (MCT: 0 g/100g; Omega-3: 0 g/100g; lard: 69 g/100g); Carbohydrate: 3 g/100g; AN-76 mineral mixture: 2.5 g/100g; AN-76 fiber mixture: 1 g/100g; Fiber: 4 g/100g. |
| Rieger^[47]^ | Fat 6.1 g/100g; Carbohydrate 55.6 g/100g; Protein 21.8 g/100g; Fiber 3.8 g/100g. | Fat 56.1 g/100g; Carbohydrate 2.9 g/100g; Protein 15.0 g/100g; Fiber: 1.7 g/100g. |
| Poff ^[12]^ | % Cal from Fat: 18; % Cal from Protein: 24; % Cal from Carbohydrate: 58. | % Cal from Fat: 89.2; % Cal from Protein: 8.7; % Cal from Carbohydrate: 2.1. |
| Maurer^[37]^ | Fat 6.1 g/100g; Carbohydrate 55.6 g/100g; Protein 21.8 g/100g; Fiber 3.8 g/100g; Ashes 5.3 g/100g. | Fat 35.5 g/100g; Carbohydrate 0.2 g/100g; Protein 13.0 g/100g; Fiber 14.8 g/100g; Ashes 2.1 g/100g. |
| Stafford^[38]^ | - | This diet consists of 8.36% protein, 0.76% carbohydrates and 78.8% fat (173.3 g/Kg casein, 586.4 g/kg cellulose, 586.4 g/kg shortening and vitamins and minerals equal to that found in normal rodent chow. |
| Augur^[50]^ | Fat 7.0 g/kg; Carbohydrate 36.4 g/kg; Protein 23.8 g/kg; Fiber 17.3 g/kg. | Fat 35.5 g/kg; Carbohydrate 0.2 g/kg; Protein 13.0 g/kg; Fiber 14.8 g/kg. |
| Zhou^[34]^ | Fat 6.9 g/kg; Carbohydrate 65 g/kg; Protein 28.1 g/kg. | Fat 80 g/kg; Carbohydrate 3.3 g/kg; Protein 16.7 g/kg. |
| Nakamura^[19]^ | Carbohydrates 16.1 g/100g; Proteins 5.0 g/100g; Lipids 1.7 g/100g; MCT 0 g/100g. | Carbohydrates 1.2 g/100g; Proteins 2.0 g/100g; Lipids 9.7 g/100g; MCT 5.4 g/100g. |

MCT: medium chain triglycerides; MKD: ketogenic diet rich in omega-3 fatty acids and MCT;

LKD: ketogenic diet rich in lard.
